# Hsa_circ_0000467 promotes colorectal cancer proliferation and stem cell characteristics by activating the TCF4/Wnt/β-catenin pathway via sponging miR-520g

**DOI:** 10.1063/5.0252083

**Published:** 2025-04-25

**Authors:** Fanggen Xu, Yujing Wang, Rongzhou Liang, Sicong Jiang

**Affiliations:** 1 Gaoxin Branch of The First Affiliated Hospital of Nanchang University, Nanchang, Jiangxi, 330038, China; 2 Affiliated Cancer Hospital of Xinjiang Medical University, Xinjiang, Urumqi, Jiangxi 330038, China; 3 Department of Plastic and Cosmetic Surgery, The Sixth People's Hospital of Dongguan, Guangdong, China

## Abstract

This study explores the role of circ_0000467 in colorectal cancer (CRC) progression and its potential as a therapeutic target. Circ_0000467 expression was analyzed using public datasets and clinical samples from 103 CRC patients. Functional assays evaluated its influence on CRC cell proliferation, migration, and stem-like properties. Molecular interactions with miR-520g and TCF4 were examined, and *in vivo* experiments assessed tumor growth. Circ_0000467 was significantly overexpressed in CRC and associated with poor prognosis. Its upregulation enhanced tumor growth, invasion, epithelial-mesenchymal transition, and stem-like characteristics by increasing key markers (CD44, EpCAM, SOX2, and Nanog). Mechanistically, circ_0000467 acted as a molecular sponge for miR-520g, leading to increased TCF4 expression and activation of the Wnt/β-catenin pathway. Silencing TCF4 or overexpressing miR-520g reversed these effects. Circ_0000467 promotes CRC progression by regulating the TCF4/Wnt/β-catenin pathway through miR-520g, highlighting its potential as a biomarker and therapeutic target for CRC.

## INTRODUCTION

Colorectal cancer (CRC) is a leading global malignancy, with incidence and mortality rates of 10.2% and 9.2%, respectively.^1^ While the 5-year survival rate for localized and regional CRC is approximately 90% and 71%, respectively, it drastically declines to 13%–14% in cases of distant metastasis.^2^ Standard treatments, including surgery, chemotherapy, and radiotherapy, are often limited by severe side effects and the development of resistance, contributing to poor patient outcomes.^3,4^ Identifying novel molecular targets is crucial for improving CRC therapy.

Recent evidence suggests that circular RNAs (circRNAs) play a vital role in CRC progression and could serve as potential therapeutic targets.^5–7^ CircRNAs are covalently closed single-stranded molecules lacking 5′ and 3′ ends, making them structurally stable and resistant to RNase R degradation.^8,9^ Among them, circ_0000467, located at *chr13:21742126-2174253* and encoded by the *SKA3* gene,^10^ has gained attention due to its oncogenic role in gastric cancer. Studies have shown that circ_0000467 is significantly overexpressed in gastric cancer and serves as an independent prognostic factor, with its silencing reducing tumor cell malignancy.^10,11^

Although circ_0000467 has been reported to be upregulated in CRC and linked to epithelial-mesenchymal transition (EMT) and migration,^12^ the precise mechanisms underlying its role in CRC progression remain unclear. In our preliminary analysis of the GSE142837 dataset from the Gene Expression Omnibus (GEO) database, we identified circ_0000467 as highly expressed in CRC patients, correlating with poor prognosis. Bioinformatics predictions indicated that circ_0000467 may function as a competing endogenous RNA (ceRNA) by sponging miR-520g, which in turn regulates transcription factor 4 (TCF4). TCF4 is a key activator of the Wnt/β-catenin signaling pathway, a critical driver of cancer stem cell characteristics.^13,14^

Based on these findings, we hypothesize that circ_0000467 promotes CRC progression by activating the Wnt/β-catenin pathway through the miR-520g/TCF4 axis. This study aims to clarify the molecular mechanisms of circ_0000467 in CRC and explore its potential as a biomarker and therapeutic target for clinical application.

## RESULTS

### Circ_0000467 was identified as a feature biomarker for CRC

**FIG. 1.**
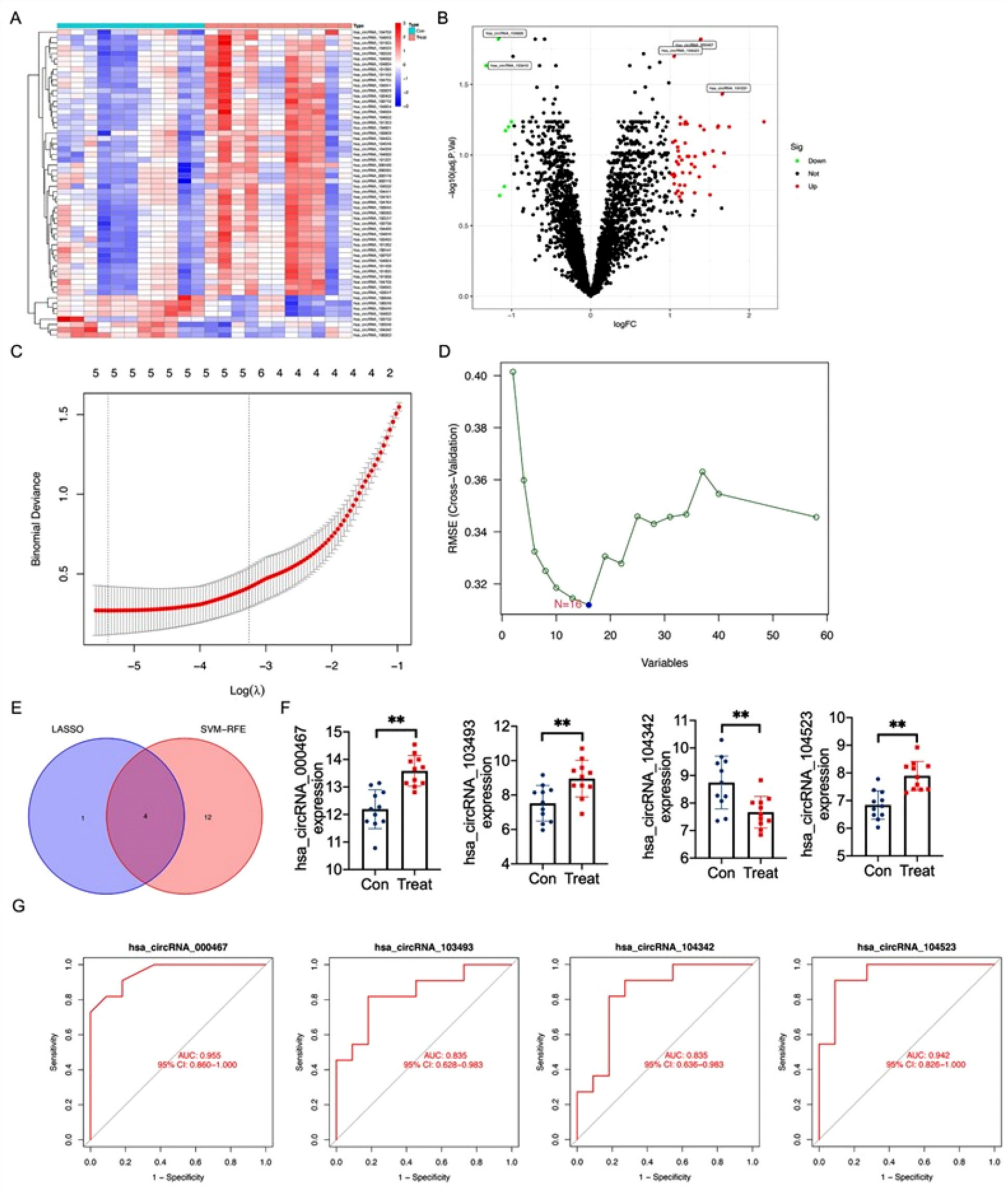
Circ_0000467 is a feature biomarker for CRC. (a) and (b) Heatmap plot showing DECs and Venn plot showing DECs based on GSE142837 and the GSE138589 datasets. (c) The selection of Tuning feature from least absolute shrinkage and selection operator (LASSO) model. (d) A plot showing biomarker screening through support vector machine-recursive feature elimination (SVM-RFE) algorithm. (e) Four diagnostic markers were selected by Venn diagram generated by the LASSO and SVM-RFE algorithms. (f) Diagnostic biomarkers were validated based on the GSE142837 and the GSE138589 datasets. (G) The diagnostic effectiveness of circ_000467, circ_103493, circ_104342, and circ_104523 was evaluated through receiver operating characteristic (ROC) curve.

This study began by screening differentially expressed circRNAs (DECs) between colorectal cancer (CRC) tumor tissues and normal tissues. A total of 58 DECs were identified, with 7 being downregulated and 51 upregulated [Figs. 1(a) and 1(b)]. Potential biomarkers were further refined using two different algorithms, and the LASSO regression algorithm reduced the number of candidates to five [Fig. 1(c)]. The SVM-RFE algorithm identified 16 potential biomarkers [Fig. 1(d)]. By comparing the results of both methods, four overlapping circRNAs—circ_000467, circ_103493, circ_104342, and circ_104523—were selected for further analysis [Fig. 1(e)]. Expression validation using the GSE142837 and GSE138589 datasets revealed that circ_000467, circ_103493, and circ_104523 were significantly upregulated, while circ_104342 was markedly downregulated in CRC tumor tissues compared to normal tissues [Fig. 1(f)]. A diagnostic model was constructed using logistic regression, and the receiver operating characteristic (ROC) curve demonstrated the diagnostic value of these circRNAs. The area under the curve (AUC) values were 0.955 (95% CI 0.860–1.000) for circ_000467, 0.835 (95% CI 0.628–0.983) for circ_103493, 0.835 (95% CI 0.636–0.983) for circ_104342, and 0.942 (95% CI 0.826–1.000) for circ_104523 [Fig. 1(g)]. Since circ_000467 exhibited the highest diagnostic ability, it was selected for further investigation in this study.

### Circ_0000467 was abnormally overexpressed in CRC

**FIG. 2.**
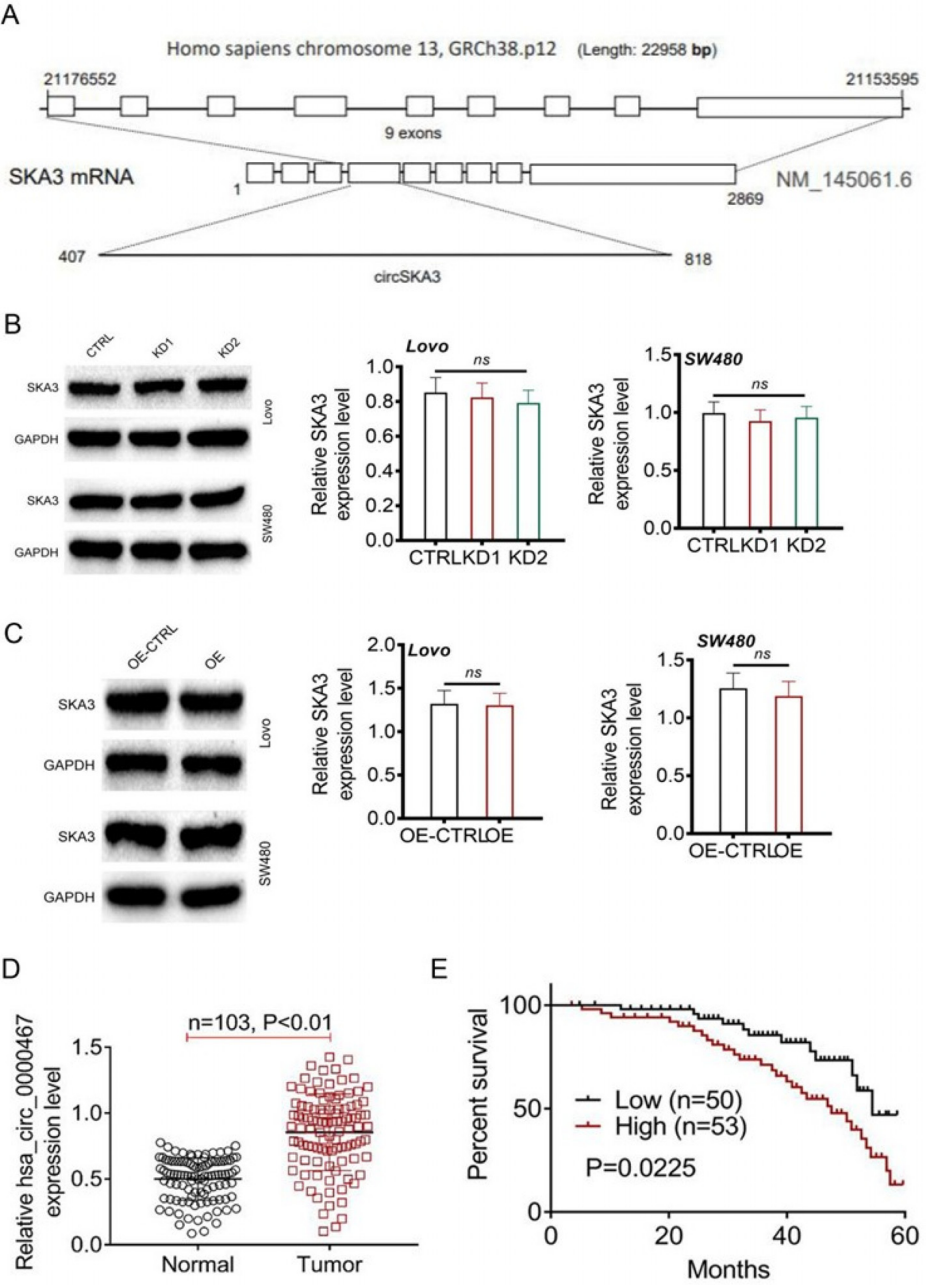
Circ_0000467 was abnormally overexpressed in CRC. (a) The SKA3 locus. (b)–(c) Knockdown of circ_000467 or circ_000467 overexpression showed no significant changes on SKA3 expression in CRC cells. (d) Circ_0000467 expression in CRC patients as detected by qRT-PCR. (e) Kaplan–Meier curve for survival analysis in CRC patients.

The SKA3 locus is illustrated in Fig. 2(a), and experimental results indicated that knockdown or overexpression of circ_000467 did not significantly affect SKA3 expression in CRC cells [Figs. 2(b) and 2(c)]. Clinical analysis revealed that circ_0000467 was significantly upregulated in CRC tissues compared to paired normal tissues (P < 0.01) [Fig. 2(d)]. Moreover, CRC patients with high circ_0000467 expression in tumor tissues exhibited worse overall survival following surgery (P = 0.0225) [Fig. 2(e)].

### Circ_0000467 overexpression exacerbated CRC cell proliferation and invasion

**FIG. 3.**
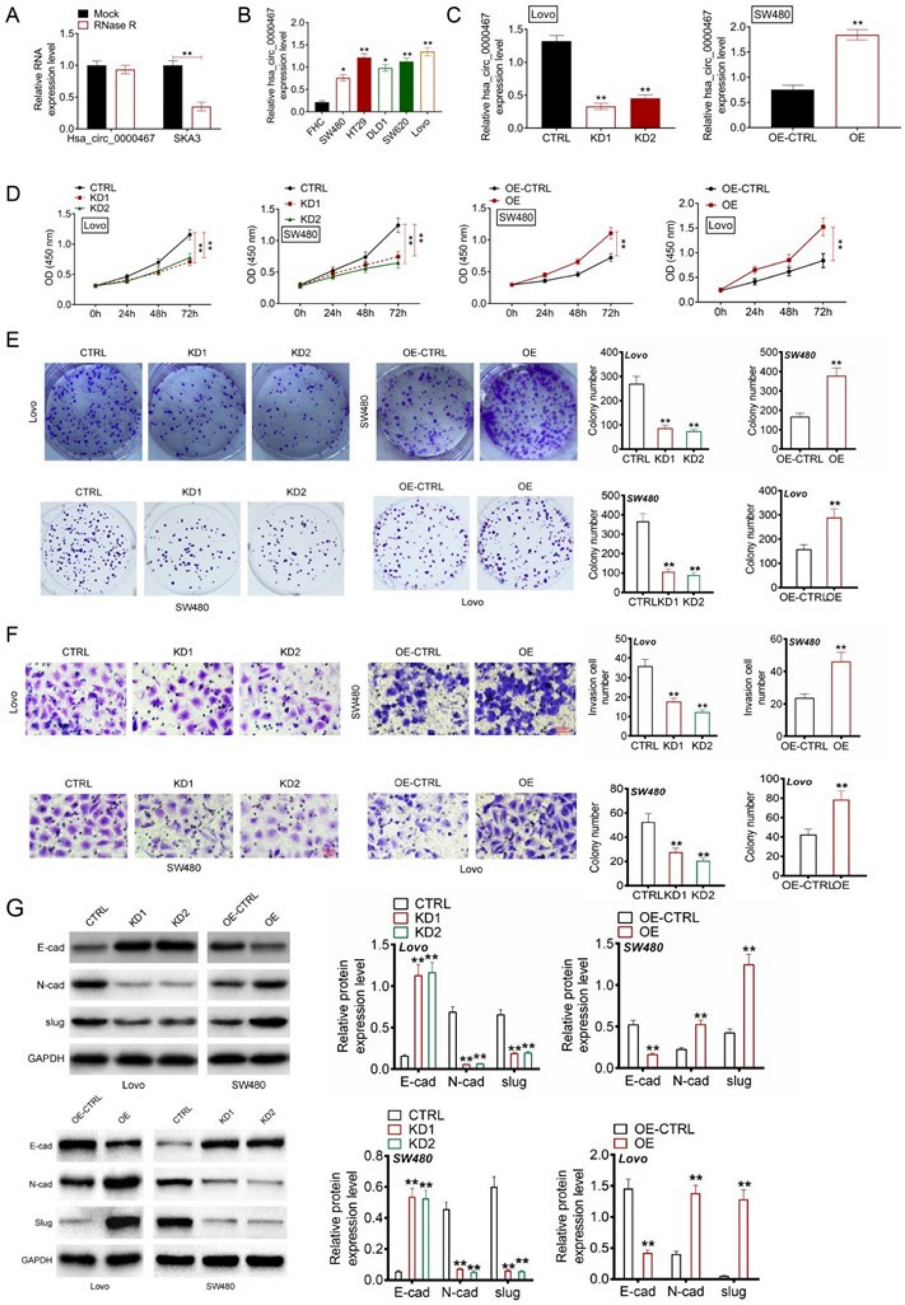
Circ_0000467 overexpression exacerbated CRC cells proliferation and invasion. (a) Circ_0000467 loop structure ability was detected by RNase R treatment. ^**^P < 0.01. (b) Circ_0000467 level in CRC cells through qRT-PCR. *P* < 0.05 or *P* < 0.01 vs FHC cell line. (c) After transfection, circ_0000467 expression within CRC cells explored via implementing qRT-PCR. (d) CRC cell proliferation as reflected through CCK-8 assay. (e) CRC cell malignant proliferation as measured through colony formation assay. (f) CRC cells invasion investigated through Transwell experiment. (g) Western blotting conducted for detecting EMT-related gene levels within CRC cells. ^**^P < 0.01 vs CTRL group or OE-CTRL group.

RNase R treatment significantly degraded SKA3 mRNA (the host gene of circ_0000467) (P < 0.01), but had no notable effect on circ_0000467 expression, suggesting a stable loop structure [Fig. 3(a)]. Compared to normal FHC cells, CRC cell lines (SW480, HT29, DLD1, SW620, and Lovo) showed markedly higher circ_0000467 expression (P < 0.05 or P < 0.01) [Fig. 3(b)]. Knockdown (KD1 and KD2 groups) and overexpression (OE group) of circ_0000467 were confirmed by qRT-PCR in Lovo and SW480 cells, demonstrating effective transfection (P < 0.01) [Fig. 3(c)].

Cell proliferation and invasion were assessed using CCK-8, colony formation, and Transwell assays [Figs. 3(d)–3(f)]. Compared to the control (CTRL) group, CRC cells with circ_0000467 knockdown exhibited significantly reduced OD values, colony numbers, and invasion ability (P < 0.01). Conversely, CRC cells with circ_0000467 overexpression displayed significantly increased proliferation and invasion (P < 0.01). Western blotting revealed that circ_0000467 knockdown led to increased E-cadherin expression and decreased N-cadherin and Slug protein levels (P < 0.01). In contrast, overexpression of circ_0000467 resulted in reduced E-cadherin expression and elevated N-cadherin and Slug protein levels (P < 0.01) [Fig. 3(g)].

### Circ_0000467 promoted CRC stem cell characteristics and growth in vivo

**FIG. 4.**
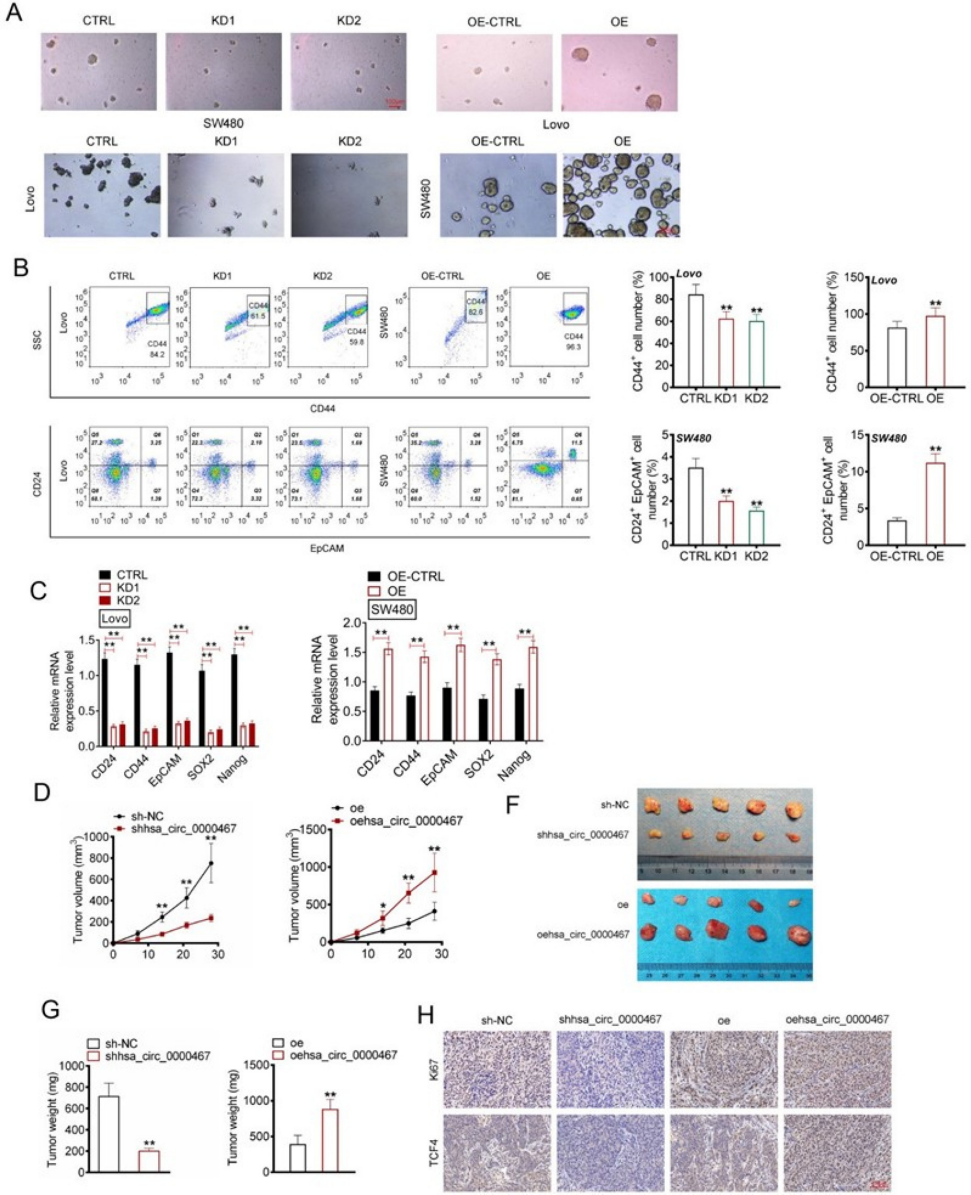
Circ_0000467 promoted CRC cells stem cell characteristics and growth *in vivo*. (a) Spheroid formation of CRC cells was investigated using spheroidization experiment. (b) CD44+ cells and CD24+/EpCAM+ cells proportion was researched through flow cytometry. *P* < 0.05 or ^**^P < 0.01 vs CTRL group or OE-CTRL group. (c) qRT-PCR was applied for CD24, CD44, EpCAM, SOX2, and Nanog mRNAs expression. ^**^P < 0.01. (d) Xenograft tumor volume in mice was measured. (e) Photos of xenograft tumor in mice. ^**^P < 0.01 vs sh-NC group or oe-NC group. (f) Mouse xenograft tumor weight was weighted. (g) IHC of xenograft tumor to detect Ki67 and TCF4 expression. ^**^P < 0.01 vs sh-NC group or oe group.

To evaluate the effect of circ_0000467 on cancer stemness, spheroid formation assays were conducted. Knockdown of circ_0000467 significantly reduced spheroid formation in Lovo and SW480 cells, while overexpression markedly increased spheroid formation in SW480 cells [Fig. 4(a)]. Flow cytometry analysis showed that knockdown of circ_0000467 resulted in fewer CD44+ and CD24+/EpCAM+ cells in Lovo cells (P < 0.01), whereas overexpression led to an increase in these stem-like populations in SW480 cells (P < 0.01) [Fig. 4(b)]. RT-PCR analysis confirmed a significant decrease in stemness-related gene expression (CD24, CD44, EpCAM, SOX2, and Nanog) in Lovo cells upon circ_0000467 knockdown (P < 0.01), while overexpression led to a marked increase in these genes in SW480 cells (P < 0.01) [Fig. 4(c)].

In vivo experiments showed that circ_0000467 knockdown significantly suppressed Lovo cell growth in mice (P < 0.01), whereas overexpression markedly promoted SW480 cell growth *in vivo* (P < 0.05 or P < 0.01) [Figs. 4(d)–4(f)]. Immunohistochemistry (IHC) analysis of xenograft tumors demonstrated that circ_0000467 knockdown significantly reduced Ki67 and TCF4-positive cells, while overexpression led to an increase in these markers [Fig. 4(g)].

### miR-520g was sponged by circ_0000467

**FIG. 5.**
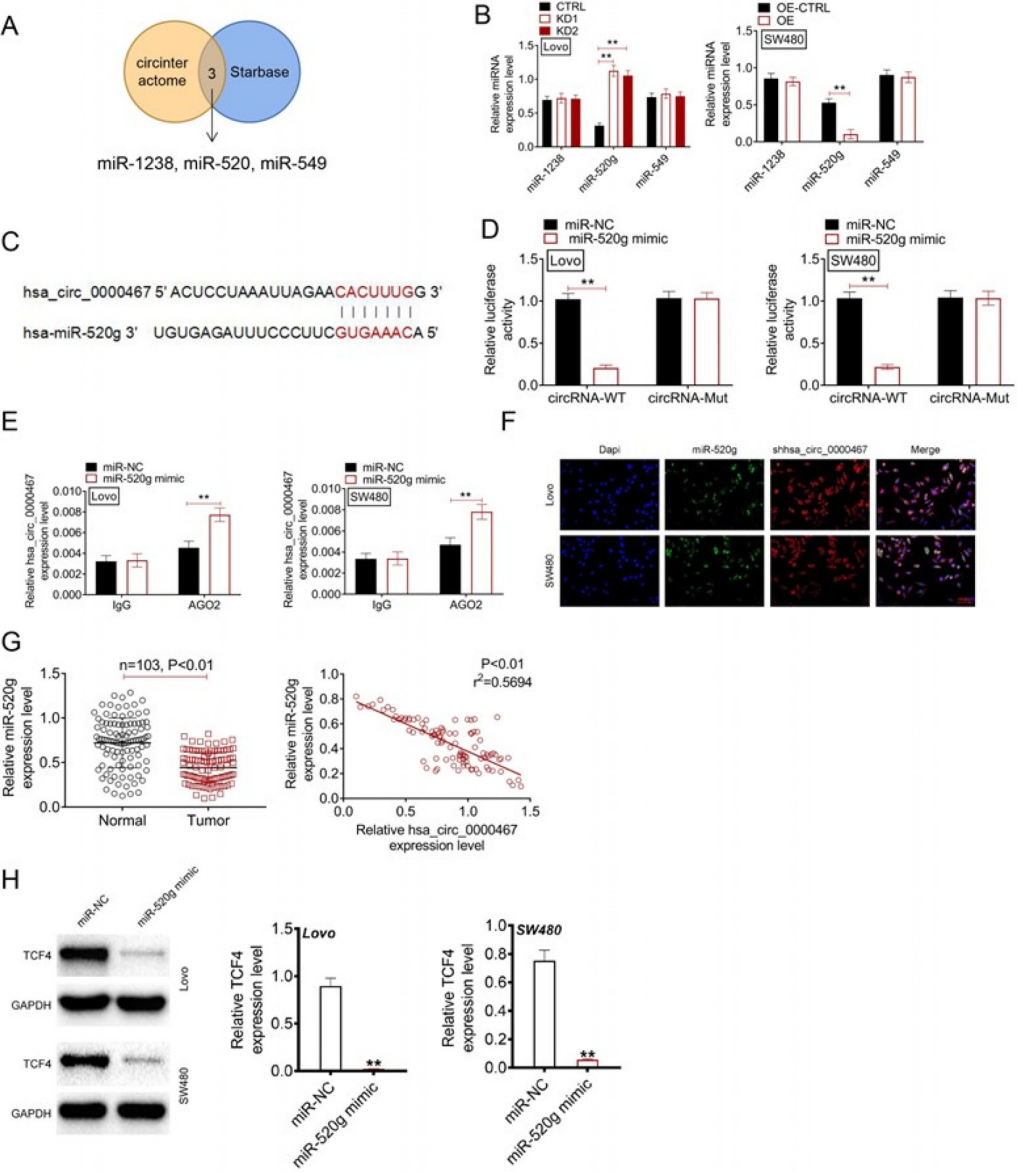
miR-520g was sponged by circ_0000467. (a) CIRCRNA website and StarBase website indicated three miRNAs containing the binding site of circ_0000467, including miR-1238, miR-520, and miR-549. (b) miR-1238, miR-520, and miR-549 expression in CRC cells as scrutinized by qRT-PCR. (c) The miR-520g binding site of circ_0000467. (d) Dual-luciferase reporter gene assay conducted to research miR-520g binding to circ_0000467. (e) RIP assay was recruited for verifying the binding relationship of miR-520g with circ_0000467. (f) RNA FISH experiment researched the co-localization of miR-520g and circ_0000467 in CRC cells. (g) miR-520g expression and its correlation with circ_0000467 in CRC patients. ^**^P < 0.01. Highlighted image legends: Functional analysis of hsa_circ_0000467 in colorectal cancer (CRC). Venn diagram identifying diagnostic markers using LASSO and SVM-RFE algorithms. Western blot analysis showing no significant changes in SKA3 expression upon circ_0000467 knockdown or overexpression. Colony formation and Transwell assays demonstrating the effects of circ_0000467 on CRC cell proliferation and invasion. qRT-PCR analysis revealing altered expression of miR-1238, miR-520g, and miR-549. Dual-luciferase reporter assay confirming miR-520g binding to circ_0000467.

Bioinformatic analysis using the CIRCRNA and StarBase databases identified three potential miRNAs (miR-1238, miR-520, and miR-549) with binding sites for circ_0000467 [Fig. 5(a)]. qRT-PCR analysis revealed that circ_0000467 knockdown significantly increased miR-520g expression in Lovo cells, whereas overexpression suppressed miR-520g levels in SW480 cells (P < 0.01). However, circ_0000467 modulation had no significant effect on miR-1238 or miR-549 expression [Fig. 5(b)], leading to the selection of miR-520g for further investigation.

Luciferase reporter assays confirmed the binding of miR-520g to circ_0000467, as overexpression of miR-520g significantly reduced the luciferase activity of the circ_0000467-WT construct (P < 0.01), but had no effect on the mutant construct [Fig. 5(d)]. RNA immunoprecipitation (RIP) assay using AGO2 antibody further validated the interaction between miR-520g and circ_0000467 in both cell lines (P < 0.01) [Fig. 5(e)]. RNA fluorescence *in situ* hybridization (FISH) assays demonstrated co-localization of miR-520g and circ_0000467 in the cytoplasm of CRC cells [Fig. 5(f)]. Clinically, miR-520g was significantly downregulated in CRC tissues compared to normal tissues (P < 0.01), and its expression negatively correlated with circ_0000467 levels (P < 0.01) [Fig. 5(g)].

### TCF4 served as the direct target of miR-520g

**FIG. 6.**
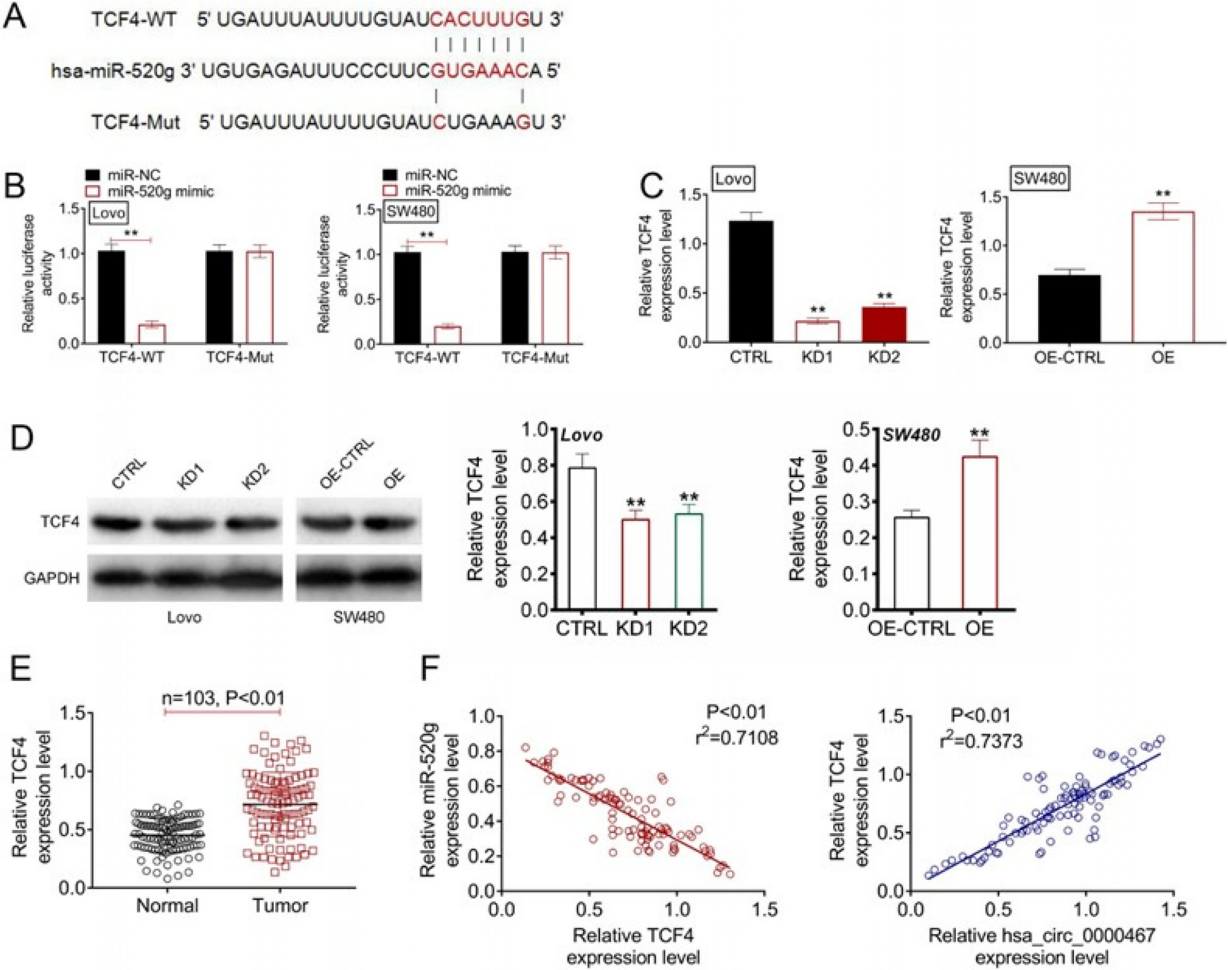
TCF4 was miR-520g's direct target. (a) miR-520g binding site of TCF4 as predicted using TargetScan. (b) Dual-luciferase reporter gene assay conducted to confirm binding between miR-520g and TCF4. ^**^P < 0.01. (c) TCF4 mRNA level within CRC cells was detected through qRT-PCR. (d) TCF4 protein expression tested through Western blotting. (e) TCF4 mRNA expression in CRC patients as explored through qRT-PCR. (f) Association of TCF4 mRNA with miR-520g or circ_0000467 expression in clinical CRC samples detected through Pearson's correlation analysis. ^**^P < 0.01 vs CTRL group or OE-CTRL group.

TargetScan analysis predicted a binding site for miR-520g within the 3'UTR of TCF4 [Fig. 6(a)]. Luciferase reporter assays revealed that miR-520g overexpression significantly reduced TCF4-WT luciferase activity in CRC cells (P < 0.01), whereas the mutant construct showed no change [Fig. 6(b)]. Knockdown of circ_0000467 significantly reduced TCF4 mRNA and protein levels in Lovo cells (P < 0.01), whereas overexpression increased TCF4 expression in SW480 cells (P < 0.01) [Figs. 6(c) and 6(d)]. Clinical analysis demonstrated that TCF4 mRNA was significantly upregulated in CRC samples (P < 0.01) and showed a positive correlation with circ_0000467 expression but a negative correlation with miR-520g levels (P < 0.01) [Figs. 6(e) and 6(f)].

### Circ_0000467 promoted CRC progression via the TCF4/Wnt/β-catenin pathway

Further experiments demonstrated that circ_0000467 promoted CRC cell proliferation and stemness by activating the TCF4/Wnt/β-catenin signaling pathway through sponging miR-520g. These findings highlight circ_0000467 as a potential biomarker and therapeutic target in CRC.

### Circ_0000467 promoted proliferation and stem cell characteristics of CRC cells via TCF4/Wnt/β-catenin pathway activation through sponging miR-520g

#### Transfection efficiency assessment

**FIG. 7.**
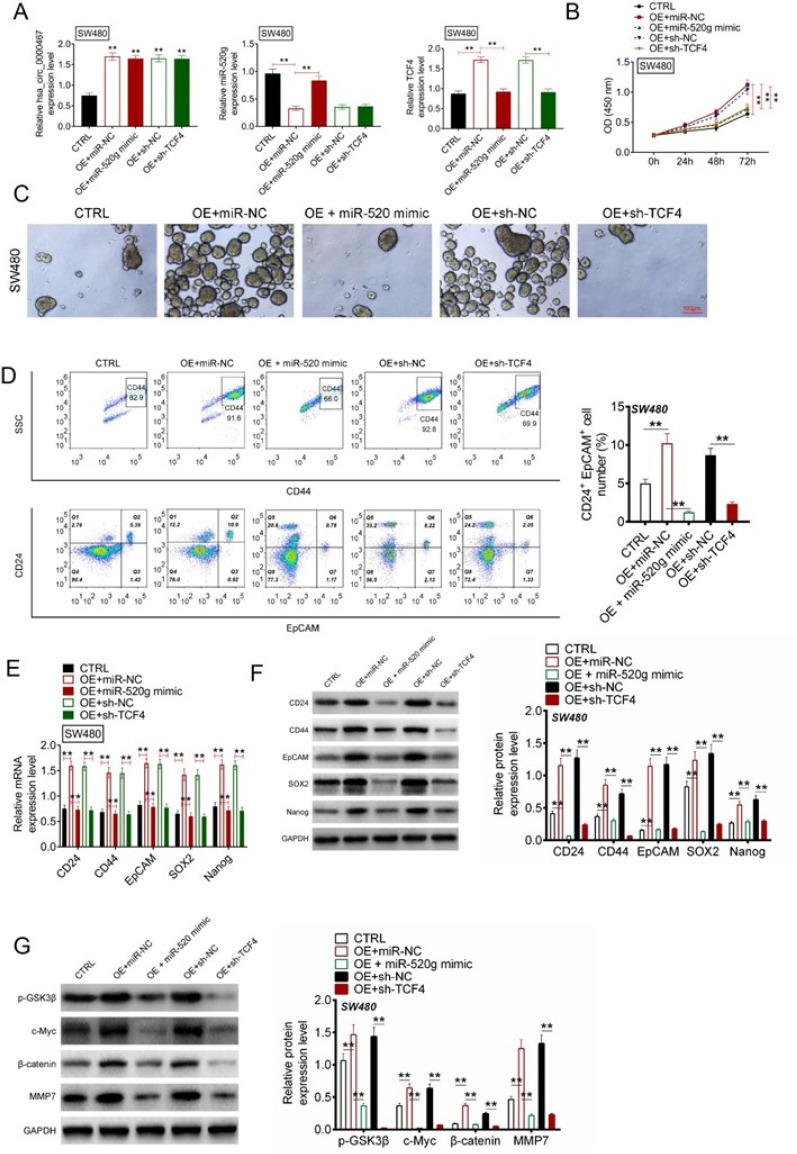
Circ_0000467 promoted proliferation and stem cell characteristics of CRC cells by sponging miR-520g/TCF4 axis. (a) SW480 cells transfection efficiency determined through qRT-PCR. (b) CCK-8 assay conducted to analyze CRC cells proliferation. (c) Spheroidization experiment was applied for spheroid formation detection of CRC cells. (d) CD24+/EpCAM+ cells proportion was researched by flow cytometry. (e) CD24, CD44, EpCAM, SOX2, and Nanog mRNAs expression determined through qRT-PCR. (f) CD24, CD44, EpCAM, SOX2, and Nanog proteins expression explored through Western blotting. (g) p-GSK3β, c-Myc, β-catenin, and MMP7 protein levels within CRC cells scrutinized through Western blotting. ^**^P < 0.01.

Following the transfection of SW480 cells, qRT-PCR was conducted to evaluate transfection efficiency. Compared to the OE-CTRL group, SW480 cells in the OE+ miR-NC, OE+ miR-520 mimic, OE+ sh-NC, and OE+ sh-TCF4 groups exhibited significantly increased circ_0000467 levels (P < 0.01). Additionally, relative to the OE+ miR-NC group, a notable upregulation of miR-520g expression was observed in the SW480 cells from the OE-CTRL and OE+ miR-520 mimic groups (P < 0.01). Regarding TCF4 mRNA expression, the SW480 cells in the OE+ miR-NC group showed a significantly higher TCF4 mRNA level compared with the OE-CTRL and OE+ miR-520 mimic groups (P < 0.01). Moreover, the SW480 cells in the OE+ sh-TCF4 group exhibited a remarkably lower TCF4 mRNA expression compared to the OE+ sh-NC group (P < 0.01) [Fig. 7(a)]. These findings confirmed the successful transfection of SW480 cells.

#### Effects on cell proliferation and spheroid formation

The CCK-8 assay and spheroidization experiments demonstrated that the SW480 cells in the OE+ miR-NC group exhibited significantly higher OD values and increased spheroid formation compared to the OE-CTRL and OE+ miR-520 mimic groups (P < 0.01). Conversely, compared to the OE+ sh-NC group, the SW480 cells in the OE+ sh-TCF4 group displayed significantly lower OD values and reduced spheroid formation (P < 0.01) [Figs. 7(b) and 7(c)].

#### Stem cell characteristics evaluation

Flow cytometry analysis revealed a significantly higher proportion of CD24^+^/EpCAM^+^ cells in the OE+ miR-NC group compared to the OE-CTRL and OE+ miR-520 mimic groups (P < 0.01). However, in comparison to the OE+ sh-NC group, the CD24^+^/EpCAM^+^ cell population significantly declined in the OE+ sh-TCF4 group (P < 0.01) [Fig. 7(d)]. qRT-PCR and Western blot analysis further confirmed that the mRNA and protein levels of CD24, CD44, EpCAM, SOX2, and Nanog were significantly increased in the OE+ miR-NC group compared to the OE-CTRL and OE+ miR-520 mimic groups (P < 0.01). In contrast, the expression of these stem cell markers was significantly reduced in the OE+ sh-TCF4 group compared to the OE+ sh-NC group (P < 0.01) [Figs. 7(e) and 7(f)].

#### Wnt/β-catenin pathway activation

Western blot analysis was used to evaluate the protein expression levels of p-GSK3β, c-Myc, β-catenin, and MMP7 in different experimental groups. The results indicated that the levels of these proteins were significantly elevated in the OE+ miR-NC group compared to the OE-CTRL and OE+ miR-520 mimic groups (P < 0.01). In contrast, the expression levels of p-GSK3β, c-Myc, β-catenin, and MMP7 were significantly decreased in the OE+ sh-TCF4 group compared to the OE+ sh-NC group (P < 0.01) [Fig. 7(g)].

## DISCUSSION

Currently, several circRNAs, including circ_0001696, circ_0043278, circ_0007534, and circ_0084615, have been identified as potential targets for colorectal cancer (CRC) treatment.^15–18^ This study highlights circ_0000467 as a novel regulator in CRC progression. Our findings indicate that high circ_0000467 expression correlates with poorer 60-month survival in CRC patients. Mechanistically, circ_0000467 promotes CRC cell proliferation and stem cell characteristics through activation of the TCF4/Wnt/β-catenin signaling pathway via miR-520g sponging. These results align with previous research, which demonstrated that circ_0000467 enhances CRC cell growth, migration, and epithelial-mesenchymal transition (EMT) via the miR-382-5p/EN2 axis.^12^ However, that study did not explore its role *in vivo*, whereas our work provides the first evidence that circ_0000467 exacerbates CRC progression *in vivo* using a xenograft tumor model. These findings establish a stronger foundation for considering circ_0000467 as a potential clinical target in CRC therapy.

Our results further show that circ_0000467 upregulates N-cadherin and Slug while downregulating E-cadherin, indicating its role in EMT—a critical process in tumor invasion and metastasis.^19^ Additionally, Ki67, a well-established marker of tumor proliferation, was significantly elevated in xenograft tumors with high circ_0000467 expression,^20^ reinforcing the notion that circ_0000467 enhances CRC cell proliferation. Furthermore, our study demonstrates that circ_0000467 promotes cancer stem cell (CSC) characteristics, as evidenced by increased spheroid formation and elevated levels of CSC markers (CD24, CD44, EpCAM, SOX2, and Nanog).^21–24^

CircRNAs are known to act as sponges for miRNAs,^25^ thereby regulating downstream gene expression.^26^ Bioinformatics analysis predicted miR-1238, miR-520, and miR-549 as potential targets of circ_0000467, but only miR-520g exhibited significant expression changes upon circ_0000467 modulation. Dual-luciferase and RIP assays confirmed the direct interaction between circ_0000467 and miR-520g. Functionally, miR-520 family members have been reported as tumor suppressors in breast cancer, lung cancer, and CRC.^27–30^ In CRC, miR-520 downregulation has been associated with increased tumor proliferation, migration, invasion, and EMT, whereas its upregulation suppresses these processes. Our findings support this, as miR-520g overexpression partially reversed the oncogenic effects of circ_0000467.^31^

Furthermore, we identified TCF4 as a direct target of miR-520g. Previous studies have demonstrated that TCF4 promotes CRC CSC characteristics.^32^ Our results reveal that TCF4 knockdown counteracts the tumor-promoting effects of circ_0000467, suggesting that circ_0000467 enhances CRC malignancy by modulating the miR-520g/TCF4 axis. TCF4, a crucial transcription factor in the Wnt/β-catenin pathway, interacts with β-catenin to drive tumor progression.^14^ In cervical cancer, TCF4 silencing suppresses proliferation and CSC characteristics by downregulating CD44 and Wnt/β-catenin pathway genes, such as β-catenin and c-Myc.^14^ Consistent with this, we observed that circ_0000467 overexpression increased β-catenin, c-Myc, MMP7, and p-GSK3β levels, whereas TCF4 knockdown had the opposite effect.^33^ Since aberrant Wnt/β-catenin signaling is a hallmark of CRC,^34^ our findings suggest that circ_0000467 contributes to CRC progression by activating this pathway via TCF4 upregulation.

## CLINICAL IMPLICATIONS

While our study provides compelling evidence of circ_0000467's oncogenic role in CRC, its clinical implications need further exploration. Given that circRNAs exhibit stability in bodily fluids, circ_0000467 could serve as a promising diagnostic or prognostic biomarker for CRC. Additionally, targeting the circ_0000467/miR-520g/TCF4 axis may provide a novel therapeutic strategy. Current CRC treatments, such as chemotherapy and targeted therapy (e.g., anti-EGFR and anti-VEGF agents), often face challenges like resistance and relapse. Integrating circRNA-targeting approaches, including antisense oligonucleotides or CRISPR-based strategies, into existing CRC therapeutic frameworks could enhance treatment efficacy.

## LIMITATIONS AND FUTURE DIRECTIONS

Despite these promising findings, our study has limitations. First, our sample size was relatively small, and patient cohorts lacked diversity, potentially limiting the generalizability of our conclusions. Larger clinical trials are necessary to validate circ_0000467 as a biomarker. Second, while we demonstrated the oncogenic effects of circ_0000467 *in vitro* and *in vivo*, the precise molecular mechanisms linking circ_0000467 to other CRC-related pathways require further investigation. Additionally, our study primarily focused on the miR-520g/TCF4/Wnt/β-catenin axis, but other potential downstream targets of circ_0000467 remain unexplored. Future studies should employ transcriptome-wide approaches to identify additional regulatory networks influenced by circ_0000467.

## CONCLUSION

In summary, our study demonstrates that circ_0000467 is upregulated in CRC and promotes tumor progression by enhancing proliferation, EMT, CSC characteristics, and *in vivo* tumor growth. Mechanistically, circ_0000467 functions as a miR-520g sponge, leading to TCF4-mediated activation of the Wnt/β-catenin pathway. These findings provide new insights into CRC pathogenesis and highlight circ_0000467 as a potential biomarker and therapeutic target. Future research should focus on validating circ_0000467's clinical relevance and exploring targeted interventions to disrupt its oncogenic effects in CRC.

## METHODS

### GEO dataset analysis

This study retrieved the GSE142837 and GSE138589 datasets from the Gene Expression Omnibus (GEO) database (http://www.ncbi.nlm.nih.gov/geo/), both containing circRNA expression profiles. The GSE142837 dataset comprised 10 samples (5 normal and 5 colorectal cancer [CRC] tumor tissues), while GSE138589 included 12 samples (6 normal and 6 CRC tumor tissues). In total, 11 normal tissue samples (control group) and 11 CRC tumor tissue samples (experimental group) were analyzed. circ_0000467 expression in CRC was visualized using volcano plots and heatmaps.

### Screening of candidate diagnostic biomarkers

To identify significant prognostic variables, we employed two machine-learning algorithms. The least absolute shrinkage and selection operator (LASSO) regression algorithm was implemented using the “glmnet” package in R to enhance prediction accuracy through regularization. Genes significantly associated with CRC classification were then identified. Additionally, the support vector machine (SVM) method, a supervised machine-learning approach commonly used for classification, was applied. Recursive feature elimination (SVM-RFE) was performed using the “e1071,” “caret,” and “kernlab” R packages to select the most discriminative genes while minimizing overfitting. The genes common to both algorithms were validated using the GSE142837 and GSE138589 datasets.

### Assessment of biomarker significance in CRC diagnosis

To evaluate the diagnostic significance of the identified biomarkers, receiver operating characteristic (ROC) curves were plotted using mRNA expression data from 11 CRC tumor and 11 normal tissue samples. The area under the curve (AUC) values were calculated to assess their diagnostic accuracy, followed by validation using the GEO datasets.

### Clinical samples

A total of 103 CRC patients who underwent surgical resection at Changhai Hospital were included in this study. Informed consent was obtained from all patients, and the study was approved by the Ethics Committee of Changhai Hospital. Paired tumor and adjacent non-carcinoma tissues were collected intraoperatively and stored at −80 °C. Patients were newly diagnosed with CRC and had not received prior cancer-related treatment. Clinical data were collected from April 2013 to January 2016, with a 60-month follow-up period until patient death or the last follow-up date.

### RNase R treatment

To assess the stability of circ_0000467, total RNA was extracted from CRC tissues using TRIzol reagent (Beyotime, Shanghai, China). Samples (3 μg total RNA) were treated with RNase R (10 U; Yanhui Biotechnology, Shanghai, China) at 37 °C for 30 min. The expression levels of circ_0000467 and its linear counterpart, SKA3 mRNA, were quantified by qRT-PCR. Untreated RNA served as a control (Mock group).

### Cell culture

Normal colonic epithelial cells (FHC; Catalogue No.: XY-1283, Xuanya Biotechnology, Shanghai, China) and CRC cell lines, including SW480 (XY-XB-1711), SW620 (XY-XB-1177), HT29 (H-HT-29), DLD1 (GOY-0399X), and Lovo (KL-0144), were obtained from biotechnology suppliers in Shanghai, China. Cells were maintained in Dulbecco's modified Eagle Medium (DMEM) supplemented with 10% fetal bovine serum (FBS) at 37 °C under 5% CO_2_.

### Cell transfection

Lovo and SW480 cells (1 × 10^6^/mL) were suspended in serum-free DMEM and seeded in 6-well plates. Transfection was performed using Lipofectamine 3000 reagent (Thermo Fisher Scientific, Waltham, MA, USA). Lovo cells were transfected with circ_0000467 siRNA#1 (KD1), siRNA#2 (KD2), or a negative control siRNA (CTRL). SW480 cells were transfected with either circ_0000467 overexpression vectors (OE) or empty vectors (OE-CTRL). Co-transfection experiments included

• circ_0000467 overexpression vectors with miR-520 mimic (OE + miR-520 mimic),
• circ_0000467 overexpression vectors with miR-520 mimic negative control (OE + miR-NC),
• circ_0000467 overexpression vectors with TCF4 shRNA (OE + sh-TCF4),
• circ_0000467 overexpression vectors with TCF4 shRNA negative control (OE + sh-NC).

All transfection reagents were obtained from GeneChem (Shanghai, China). Transfections were carried out for 8 h at 37 °C and 5% CO_2_, followed by incubation in fresh DMEM (containing 10% FBS) for 48 h.

### Cell proliferation assay (CCK-8)

Cell proliferation was assessed using the Cell Counting Kit-8 (CCK-8) assay. Lovo and SW480 cells (1 × 10^4^ cells/well) were seeded in 96-well plates and cultured for 0, 24, 48, and 72 h. After incubation, CCK-8 solution was added, and optical density (OD) at 450 nm was measured using a microplate reader (Thermo Labsystems, Franklin, MA, USA). Each group had five replicates.

### Colony formation assay

To assess malignant cell proliferation, 1 × 10^3^ Lovo or SW480 cells were plated in 6-well plates with 10% FBS-containing DMEM and cultured for 2 weeks. The medium was refreshed every 3 days. Colonies were fixed with 4% paraformaldehyde and stained with 0.1% crystal violet. Colonies containing at least 50 cells were counted under a microscope (Oberkochen, Baden-Württemberg, Germany). Experiments were performed in triplicate.

### Transwell invasion assay

Cell invasion was evaluated using Transwell chambers (pore size: 8 μm) pre-coated with Matrigel (Boster, Wuhan, China). Lovo and SW480 cells (1 × 10^4^) were seeded in the upper chambers with serum-free DMEM, while 10% FBS-containing DMEM was placed in the lower chambers. After 24 h at 37 °C and 5% CO_2_, non-invading cells were removed, while invaded cells on the lower membrane surface were fixed, stained with crystal violet, and counted under a microscope. Each group was tested in triplicate.

### Spheroid formation assay

Lovo and SW480 cells (1 × 10^3^) were cultured in 10% FBS-containing DMEM in ultra-low attachment 35 mm dishes for 10 days at 37 °C with 5% CO_2_. The medium was refreshed every 3 days. Spheroid formation was observed and imaged under a microscope.

### Flow cytometry

The proportion of CD44^+^ and CD24^+^/EpCAM^+^ cells was analyzed using flow cytometry. Lovo and SW480 cells (1 × 10^5^) were washed with PBS and incubated with fluorescently labeled antibodies: CD44-allophycocyanin, CD24-phycoerythrin, and EpCAM-fluorescein isothiocyanate (Boster, Wuhan, China). After 30 min, cells were washed, resuspended in PBS, and analyzed using a FACSCalibur flow cytometer (BD Biosciences, San Jose, CA, USA). Flow cytometry data were processed using FlowJo software (TreeStar, San Carlos, CA, USA).

### Dual-luciferase reporter assay

Potential miRNA binding sites for circ_0000467 and TCF4 were predicted using the CIRCRNA, StarBase, and TargetScan databases, identifying miR-520g as a target. Wild-type (WT) and mutant (Mut) sequences of circ_0000467 and TCF4 were cloned into luciferase reporter vectors (Promega, Madison, WI, USA). Co-transfection combinations included

• miR-520g mimic + circRNA-WT,
• miR-520g mimic + circRNA-Mut,
• miR-520g mimic + TCF4-WT,
• miR-520g mimic + TCF4-Mut.

Luciferase activity was measured 48 h post-transfection using a dual-luciferase reporter assay system (Promega).

#### RNA immunoprecipitation (RIP) assay

The miR-520g mimic- and miR-NC-transfected Lovo and SW480 cells (1 × 10^7^ cells) were treated by 100 μL of RIP lysis buffer (Solarbio, Beijing, China). We later probed 200 μL cell lysates using antibody against Ago2- or IgG-coated magnetic beads. The mixed solution was rotated for 12 h under 4 °C. Then, the immunoprecipitated RNA extraction was implemented by applying RNeasy MinElute Cleanup Kit (Qiagen, Valencia, CA, USA). Then, Reverse transcription kit (Applied Bio-systems, Foster City, CA, USA) was adopted for reverse transcription. The immunoprecipitated miR-520g and circ_0000467 levels were quantified through qRT-PCR.

#### RNA fluorescence in situ hybridization (FISH) assay

About 1 × 10^5^ Lovo and SW480 cells (without transfection) within 1 ml of 10% FBS-containing DMEM were cultivated into 6-well plates under 37 °C with 5% CO_2_ for a 24-h period. Alexa Fluor 488-labeled miR-520g as well as Alexa Fluor 594-labeled circ_0000467 probes were provided by RiboBio (Guangzhou, China). According to manufacturer's directions, Fluorescent in Situ Hybridization Kit (RiboBio, Guangzhou, China) was recruited for positive probe signals detection. 4′,6-diamidino-2-phenylindole (Dapi) was used for nuclear staining. The fluorescence microscope (Zeiss, Oberkochen, Germany) was adopted for cell observation.

#### qRT-PCR

We extracted total tissue and cellular RNAs based on the TRIzol kit instructions (Beyotime, Shanghai, China). cDNA templates were obtained by reverse transcription with the Reverse transcription kit (Applied Bio-systems, Foster City, CA, USA). cDNA templates were qualified by applying the ABI StepOne™ Real-Time PCR Systems (Applied Bio-systems, Carlsbad, CA, USA) using the following parameters: 3 min under 95 °C, 15 s under 95 °C, and 30 s under 60 °C. This program was repeated 40 times. U6 and GAPDH were references for miRNAs and circ_0000467 as well as mRNAs separately. The miRNAs, circ_0000467, and mRNAs expression was evaluated by 2^−ΔΔCT^ approach. Primer details are provided in Table I.

**TABLE I.** Primers details are provided below which were used in study.

Gene/miRNA	Forward (F) sequence	Reverse (R) sequence
Circ_0000467	5′‐AATGGGACTTAAAAATGCGAGG‐3′	5′‐GTTGTGGACTACGTGGAGACT‐3′
CD24	5′‐GTCTTTTGTTCGCATGGTC‐3′	5′‐TTCGATCTGTTTGTTCCCA‐3′
CD44	5′‐AGTCCCTGGATCACCGA‐3′	5′‐CCTCTTGGTTGCTGTCTCA‐3′
EpCAM	5′‐GTTCGGGCTTCTGCTTG-3′	5′‐ACGGCCAGCTTGTAGTTTT-3′
SOX2	5′‐CGAACCATCTCTGTGGTCT-3′	5′‐GTGTCAACCTGCATGGC-3′
Nanog	5′‐CCCTGATTCTTCCACCAGT-3′	5′‐CGGGACCTTGTCTTCCTT-3′
miR-1238	5′‐GGGCTCCCTCATCTGTC-3′	5′‐GTTGTGGTTGGTTGGTTTGT-3′
miR-520g	5′‐GGGACAAAGTGCTTCCCTTTA-3′	5′‐CAGGTTCAGGTTCAGGTTCA-3′
miR-549	5′‐AGGCGTGACAACTATGGATG-3′	5′‐GAGAGGAGAGGGAGAGGAGA-3′
TCF4	5′‐GACCACACGAACAACAGCTT-3′	5′‐TCTTCGATTCGGCTTTGCAG-3′
GAPDH	5′‐GGTCGGAGTCAACGGATTTG‐3′	5′‐ATGAGCCCCAGCCTTCTCCAT‐3′
U6	5′‐CTCGCTTCGGCAGCACA‐3′	5′‐AACGCTTCACGAATTTGCGT‐3′

#### Western blotting

Extraction of total proteins in Lovo and SW480 cells was conducted following the manufacturer's directions of RIPA protein extraction reagent (Kemin Biotechnology, Shanghai, China). Total protein contents were assessed by BCA kit (Lianmai Biological Engineering, Shanghai, China) based on specific protocols. Protein aliquots (30 *μ*g) were separated via 10% sodium dodecyl sulfate-polyacrylamide gel electrophoresis, before transfer on polyvinylidene fluoride (PVDF) membranes and 2-h blocking by 5% defatted milk. Subsequently, rabbit anti-primary antibodies (1:1000) were used for probing PVDF membrane overnight under 4 °C, including CD24 (GD-Ab013141086, Guidechem, Shanghai, China); anti-p-GSK3β (AF1531, Beyotime, Shanghai, China); anti-E-cad (ab15148), anti-N-cad (ab18203), anti-slug (ab106077), anti-TCF4 (1:1000, ab185736), anti-CD44 (ab157107), anti-EpCAM (ab71916), anti-SOX2 (ab97959), anti-Nanog (ab21624), anti-c-Myc (ab32072), anti-β-catenin (ab16051), anti-MMP7 (ab207299), and anti-GAPDH (ab9485, Abcam, Shanghai, China). Then, the PVDF membrane was treated for 2 h using horseradish peroxidase-labeled goat anti-rabbit secondary antibody (1:2000, ab6721, Abcam, Shanghai, China) at ambient temperature. Afterward, this study applied enhanced chemiluminescence (ECL) reagent to develop specific protein blots. ImageJ software (National Institutes of Health, Bethesda, MD, USA) was employed in protein quantification, with GAPDH as control.

#### In vivo experiment

We obtained twenty 4-week-old BALB/C nude mice in SIPPR-BK Laboratory Animal Company (Shanghai, China) and raised within the (22 ± 1) °C room with 12-h/12-h day/night cycle. Animals ate food and drunk water freely. The Animal Ethics Committee of Changhai Hospital approved our animal experimental protocols, following Guide for the Care and Use of Laboratory Animals. circ_0000467 shRNA and negative control were individually transfected into Lovo cells. Further, we transfected SW480 cells with circ_0000467 overexpression vectors and empty vectors, then, these cells were collected to prepare cell suspension using PBS (1 × 10^7^ ml). The 20 animals were randomized as four groups: shhsa_circ_0000467 group), sh-NC group, oehsa_circ_0000467 group and oe-NC group, with five mice group. Mice of shhsa_circ_0000467 and sh-NC groups were given subcutaneous injection of Lovo cells following circ_0000467 shRNA and corresponding NC transfection. Similarly, mice of the oehsa_circ_0000467 group and oe-NC group were given injection of SW480 cells after transfection with circ_0000467 overexpression vectors and empty vectors. Cell suspension (100 *μ*L) was injected. Xenograft tumor was measured at 1-week interval as follows, (length × width^2^)/2. Mouse euthanasia was completed 4 weeks later, and then xenograft tumor in each mouse was striped. Xenograft tumor was photographed and weighted. Thereafter, xenograft tumor was preserved under -80 °C.

#### Immunohistochemistry (IHC)

Xenograft tumor was embedded in paraffin before being prepared in 4-*μ*m sections. Sections experienced deparaffinage and rehydration via using xylene and gradient alcohol in turn. 10-min treatment of 3% H_2_O_2_ was conducted to remove endogenous peroxidase. After being boiled for 3 min in sodium citrate buffer (pH6.0), normal goat serum was added to block sections for 30 min under 37 °C. Primary antibodies (1:100, Abcam, Shanghai, China), including rabbit anti-Ki67 (ab15580) and anti-TCF4 (ab185736), were introduced for 12-h treatment under 4 °C. Goat anti-rabbit secondary antibody (1:200, ab205718) was introduced for another 1-h sections treatment under ambient temperature. Color reaction was then carried out with DAB and hematoxylin. Gradient alcohol and xylene were used sequentially for dehydration and transparency. Finally, neutral resin was added to close sections before being photographed with the microscope (Oberkochen, Baden, Wurttemberg, Germany).

#### Statistical analysis

Data represented by mean ± standard deviation were examined by GraphPad Prism 5 software (GraphPad Software Inc., San Diego, CA, USA). Pairwise comparison in both groups was examined through paired Student's t test (two-tailed). Differences across multiple groups were assessed through one-way analysis of variance (ANOVA) and Tukey's post hoc test. Correlation analysis of gene expression was examined through Pearson's correlation. *P* < 0.05 stood for statistical significance.

## Data Availability

The data that support the findings of this study are available from the corresponding author upon reasonable request.
